# The Combined Metabolically Oriented Effect of Fucoidan from the Brown Alga *Saccharina cichorioides* and Its Carboxymethylated Derivative with 2-Deoxy-D-Glucose on Human Melanoma Cells

**DOI:** 10.3390/ijms241512050

**Published:** 2023-07-27

**Authors:** Olesya S. Malyarenko, Roza V. Usoltseva, Artem S. Silchenko, Anastasiya O. Zueva, Svetlana P. Ermakova

**Affiliations:** G.B. Elyakov Pacific Institute of Bioorganic Chemistry, Far Eastern Branch, Russian Academy of Sciences, Pr. 100-Letiya Vladivostoka 159, 690022 Vladivostok, Russia

**Keywords:** *Saccharina cichorioides*, fucoidan, carboxymethylated fucoidan, 2-deoxy-D-glucose, melanoma, SK-MEL-28 cells, glycolysis

## Abstract

Melanoma is the most aggressive and treatment-resistant form of skin cancer. It is phenotypically characterized by aerobic glycolysis that provides higher proliferative rates and resistance to cell death. The glycolysis regulation in melanoma cells by means of effective metabolic modifiers represents a promising therapeutic opportunity. This work aimed to assess the metabolically oriented effect and mechanism of action of fucoidan from the brown alga *Saccharina cichorioides* (ScF) and its carboxymethylated derivative (ScFCM) in combination with 2-deoxy-D-glucose (2-DG) on the proliferation and colony formation of human melanoma cell lines SK-MEL-28, SK-MEL-5, and RPMI-7951. The metabolic profile of melanoma cells was determined by the glucose uptake and Lactate-Glo^TM^ assays. The effect of 2-DG, ScF, ScFCM, and their combination on the proliferation, colony formation, and activity of glycolytic enzymes was assessed by the MTS, soft agar, and Western blot methods, respectively. When applied separately, 2-DG (IC_50_ at 72 h = 8.7 mM), ScF (IC_50_ at 72 h > 800 µg/mL), and ScFCM (IC_50_ at 72 h = 573.9 μg/mL) inhibited the proliferation and colony formation of SK-MEL-28 cells to varying degrees. ScF or ScFCM enhanced the inhibiting effect of 2-DG at low, non-toxic concentrations via the downregulation of Glut 1, Hexokinase II, PKM2, LDHA, and pyruvate dehydrogenase activities. The obtained results emphasize the potential of the use of 2-DG in combination with algal fucoidan or its derivative as metabolic modifiers for inhibition of melanoma SK-MEL-28 cell proliferation.

## 1. Introduction

Melanoma is a highly aggressive form of skin cancer with an increasing prevalence worldwide [1]. In 2020, an estimated 325,000 new cases of melanoma were diagnosed all over the world, and 57,000 people died from the disease [2]. Even with recent advancements in melanoma therapies, namely, immunotherapies, such as ipilimumab, targeted therapies, such as vemurafenib, or combination therapies, such as polychemotherapy, polyimmunotherapy, and biochemotherapy, the management of advanced melanoma is very challenging [3,4,5,6]. The highly proliferative and invasive phenotypes of melanoma cells exhibit an increased demand for energy and building blocks and, consequently, reprogramming of cellular metabolism [7]. The malignant state of melanoma is associated with higher glycolytic activity and utilization of glucose and with lower mitochondrial respiration, even under normoxic conditions [8,9]. This metabolic profile is known as the Warburg effect and is a hallmark of many cancer types [10]. Therefore, interfering with the metabolism of melanoma cells may be a promising effective therapeutic strategy that may help to improve the existing standard therapies.

2-Deoxy-D-glucose (2-DG) is a well-known metabolic modifier that targets glucose metabolism to deplete cancer cells of energy. It was reported to compete with glucose for hexokinase-mediated phosphorylation to form 2-DG-6-phosphate, which is not further metabolized to any significant extent [11,12,13]. However, 2-DG itself has limited therapeutic effects in many types of cancers, and therefore, it may be combined with other potential agents to exhibit a synergistic anticancer effect.

As was repeatedly reported, sulfated polysaccharides, referred to as fucoidans and naturally occurring in brown algae, inhibit the proliferation, colony formation, and migration of various types of cancer cells, which makes them potentially effective metabolic modifiers for cancer treatments [14,15,16,17,18,19,20]. It has been shown that chemical derivatives of fucoidans from brown algae, such as oversulfated, aminated, phosphorylated, and acetylated fucoidans, possess more pronounced functional properties than native polysaccharides [21,22]. A search for the relevant literature on the carboxymethylation of polysaccharides from brown algae has shown that only data on the modification of fucoidan from the brown alga *Saccharina japonica* and studies on its antioxidant activity are currently available [23]. To the best of our knowledge, there is a lack of data on the carboxymethylation of algal fucoidan and investigations of their metabolically oriented effect against melanoma cells.

In the present study, we aimed to assess the metabolically oriented effect of fucoidan from the brown alga *S. cichorioides* and its carboxymethylated derivative in combination with 2-DG on the proliferation and colony formation of a human melanoma cell lines (SK-MEL-28, SK-MEL-5, and RPMI-7951) and elucidate the molecular mechanism of their combined action.

## 2. Results

### 2.1. Fucoidan from the Brown Alga S. cichorioides and Its Modification by Carboxymethylation

Fucoidan (ScF) was isolated from the Far Eastern brown alga *S. cichorioides* by an individual isolation scheme, including extraction with dilute hydrochloric acid and anion exchange chromatography on a Macro-Prep DEAE as described in our previous study [24]. We determined the structural characteristics of the obtained fucoidan (monosaccharide composition; molecular weight (Mw); content of sulfate and acetate groups; impurities of proteins; polyphenols) by classical chemical methods, size-exclusion chromatography, and NMR spectroscopy. An analysis of the monosaccharide composition and the degree of sulfation showed that ScF is a highly sulfated (35%) fucan (Table 1).

It was previously found that *S. cichorioides* produces predominantly fucoidan with a backbone of mainly (1→3)-linked α-L-fucopyranose residues and a small amount of (1→4)-linked fucopyranose and branches at position 2 in the form of single α-L-fucose residues. Sulfate groups were found at positions 2 and 4 [25]. An NMR spectroscopy confirmed that the ScF obtained in the present study had a similar structure (Supplementary Figure S1a).

Modification of polysaccharides is one of the promising ways of obtaining new compounds for medicine, chemistry, ecology, and industry. In the present study, the carboxymethylation of ScF was based on the reaction of the O-alkylation of the polysaccharide with acetic acid in NaOH and isopropanol.

We studied the structure of the obtained polysaccharide, ScFCM, via ^13^C NMR spectroscopy (Supplementary Figure S1b). It confirmed that this derivative contained carboxymethyl groups. Thus, the signals belonging to the carbonyl group at 178.8 ppm and to the methylene carbon at 71.1 ppm appeared in the spectrum after the modification (Supplementary Figure S1b). The determined structural characteristics of the carboxymethylated derivative of ScF, ScFCM, used in this study are presented in Table 1.

### 2.2. The Effect of Fucoidan from S. cichorioides and Its Carboxymethylated Derivative on the Glucose Uptake and the Lactate and Glutamate Production in Human Melanoma Cells

In this study, we determined the metabolic profile of normal human immortal keratinocyte cells (HaCaT) and human melanoma cells (SK-MEL-28, SK-MEL-5, and RPMI-7951) by their ability to uptake glucose induced by insulin or excrete lactate and glutamate.

**Figure 1:**
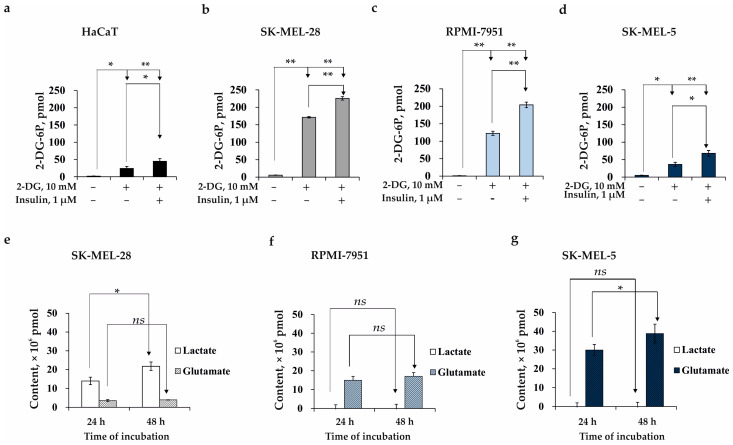
Metabolic profile of HaCaT, SK-MEL-28, RPMI-79, and SK-MEL-5 cells. The ability of HaCaT cells (**a**), SK-MEL-28 cells (**b**), RPMI-7951 (**c**), and SK-MEL-5 (**d**) to take up 2-DG as determined by Glucose Uptake Colorimetric Assay. The ability of SK-MEL-28 (**e**), RPMI-7951 (**f**), and SK-MEL-5 (**g**) cells to excrete the lactate and glutamate as determined by Lactate/Glutamate Glo assay. Data show the mean of three independent experiments ± SD. A one-way ANOVA and Tukey’s HSD test for multiple comparisons indicated the statistical significance (*ns*—no significant difference, * *p* < 0.05, ** *p* < 0.01).

We found that the HaCaT cells took up 24 pmol of 2-deoxy-glucose (2-DG) without insulin stimulation or 45 pmol of 2-DG induced by insulin, indicating a moderate ability of this cell type to take up glucose (Figure 1a). The SK-MEL-28 and RPMI-7951 cells took up 172 pmol and 123 pmol of 2-DG without insulin, respectively, while the insulin stimulation induced an increase in glucose uptake by 230 and 204 pmol, respectively, which is typical for cells of a glycolytic metabolic profile (Figure 1b,c). In contrast, SK-MEL-5 cells were able to take up 36 or 68 pmol of glucose without or with insulin stimulation, respectively (Figure 1d).

To confirm the preferred metabolic profile of the cancer cells tested, we also estimated the lactate and glutamate production. Since the lactate produced by glycolysis is released from the cell and the glutamate produced by oxidative phosphorylation is accumulated within the cell, we analyzed both cell lysates and cell culture media. As a result, the amount of lactate in the culture medium of SK-MEL-28 melanoma cells reached 13.98 × 10^6^ and 21.75 × 10^6^ pmol, while the amount of lactate produced by RPMI-7951 or SK-MEL-5 cells was not significant (Figure 1e–g). The amount of glutamate in the cell lysates of SK-MEL-28, RPMI-7951, and SK-MEL-5 was 3.61 × 10^6^ and 3.96 × 10^6^ pmol; 11.91 × 10^6^ and 15.46 × 10^6^ pmol; and 30.01 × 10^6^ and 38.80 × 10^6^ pmol at 24 and 48 h, respectively (Figure 1e–g).

The results obtained provided evidence that normal human immortal keratinocyte cells (HaCaT) are characterized by the modest ability to take up glucose and produce lactate and glutamate, which is inherent in normal cells. The glycolysis metabolic pathway with high lactate excretion is preferable only for SK-MEL-28 cells among the tested melanoma cells (Figure 1b,e). RPMI-7951 cells were able to take up a high amount of glucose but produced fewer amounts of lactate and glutamate (Figure 1c,f). SK-MEL-5 was determined to excrete a high amount of glutamate that was characteristic of cells predominantly with mitochondrial oxidative phosphorylation (OXPHOS) metabolic profile (Figure 1d,g).

**Figure 2:**
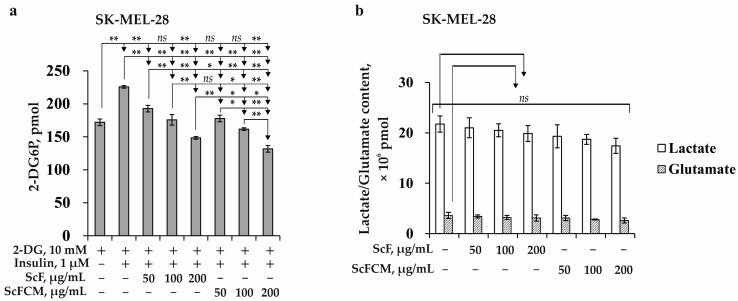
The effect of fucoidan and its carboxymethylated derivative on glucose uptake and lactate and glutamate production in SK-MEL-28 melanoma cells. The effect of fucoidan from *S. cichorioides* (ScF) and its carboxymethylated derivative (ScFCM) on (**a**) the insulin-stimulated uptake of glucose and (**b**) the lactate and glutamate production. Data show the mean of three independent experiments ± SD. A one-way ANOVA and Tukey’s HSD test for multiple comparisons indicated the statistical significance (*ns*—no significant difference, * *p* < 0.05, ** *p* < 0.01).

In the present study, we assessed the effect of ScF from *S. cichorioides* and ScFCM on glucose uptake and lactate and glutamate production in SK-MEL-28 cells that have a glycolytic metabolic profile (Figure 2a,b). We found that ScF at concentrations of 50, 100, and 200 µg/mL inhibited the insulin-stimulated uptake of 2-DG in SK-MEL-28 cells by 16, 22, and 34%, respectively, compared to PBS-treated cells stimulated with insulin (control) (Figure 2a). ScFCM at 50, 100, and 200 µg/mL had a more potent inhibiting effect on glucose uptake and reduced the amount of 2-DG in SK-MEL-28 cells by 21, 28, and 42%, respectively, compared to the control (Figure 2a). ScF (at 50, 100, and 200 μg/mL) had a slight effect on the lactate and glutamate production in SK-MEL-28 cells; the percentage of inhibition was less than 15% compared to the control (Figure 2b). ScFCM (at 50, 100, and 200 μg/mL) decreased the lactate content by 11, 14, and 20%, respectively, and the glutamate production in SK-MEL-28 cells by 14, 22, and 28%, respectively (Figure 2b). Thus, ScF and ScFCM were shown to possess a comparable inhibiting effect on glucose uptake and slightly influenced the lactate/glutamate production in SK-MEL-28 cells.

### 2.3. The Metabolically Oriented Effect of Fucoidan from S. cichorioides and Its Carboxymethylated Derivative on Viability and Proliferation of Human Melanoma Cells

In our study, we tested the idea that the fucoidan from the brown alga *S. cichorioides* or its carboxymethylated derivative in combination with a low dose of 2-DG would synergistically delay the glycolysis of SK-MEL-28 melanoma cells to result in pronounced inhibition of cells’ viability and proliferation.

At the first stage of bioactivity investigations, we assessed the individual effect of 2-DG (0.1–20 mM), ScF (100–800 μg/mL), and ScFCM (100–800 μg/mL) on the viability and proliferation of SK-MEL-28 cells in order to calculate their half-maximal inhibitory concentration (IC_50_) and select the effective concentrations for combinatorial treatment.

**Figure 3:**
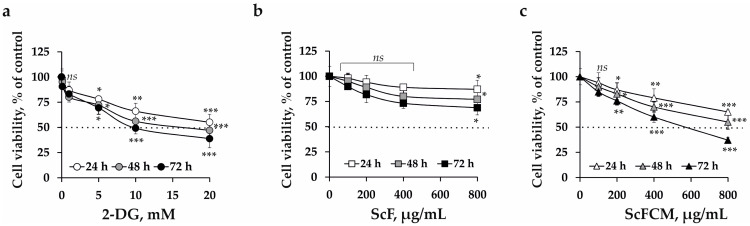
The effect of 2-DG, the fucoidan (ScF) from *S. cichorioides*, and its carboxymethylated derivative (ScFCM) on the viability and proliferation of human melanoma cells (SK-MEL-28). SK-MEL-28 cells were treated by (**a**) 2-DG (0.1–20 mM), (**b**) ScF (100–800 µg/mL), and (**c**) ScFCM (100–800 µg/mL) and incubated for 24, 48, and 72 h, respectively. The cell viability was estimated by the MTS assay. The data are presented as mean ± SD for triplicate experiments. The asterisk (*) indicates a significant decrease in cell viability of SK-MEL-28 cells treated with different concentrations of investigated compounds at a time point of 24 or 48 or 72 h as compared to control (*ns*—no significant difference, * *p* < 0.05, ** *p* < 0.01, *** *p* < 0.001).

The IC_50_ for SK-MEL-28 cells treated with an inhibitor of glycolysis 2-DG was estimated at 8.7 mM after 72 h of cells incubation (Figure 3a). The IC_50_ for fucoidan ScF was not distinguished at concentrations up to 800 μg/mL. ScF at 100, 200, 400, and 800 μg/mL inhibited the viability of SK-MEL-28 cells by 5, 12, 17, and 24%, respectively, at 72 h of cell incubation (Figure 3b), while the IC_50_ of ScFCM for SK-MEL-28 cells was 573.9 μg/mL at 72 h (Figure 3c). It should be noted that 2-DG (IC_50_ = 18.6 mM), ScF (IC_50_ > 800 μg/mL), and ScFCM (IC_50_ = 626.7 μg/mL) slightly influenced the viability of normal immortal keratinocyte cells (HaCaT) at 72 h of cells treatment (Supplementary Figure S2a–c).

To assess the metabolically oriented effect of ScF and ScFCM with 2-DG in melanoma cell treatment, we used the 2-DG concentrations of 1 mM and polysaccharide concentrations of 50, 100, and 200 μg/mL, at which they alone slightly influenced the viability and proliferation of normal cells HaCaT (Supplementary Figure S2d,e) and melanoma cells SK-MEL-28 even at 72 h of treatment (Figure 3a–c).

**Figure 4:**
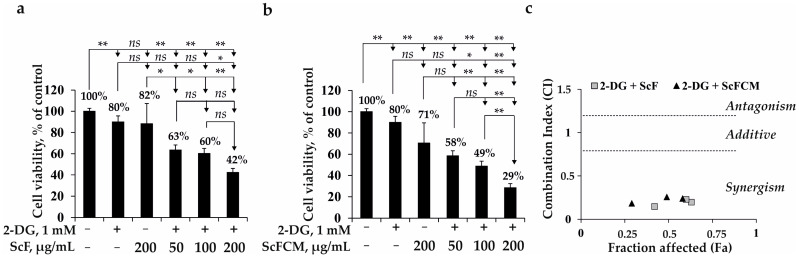
The metabolically oriented effect of 2-DG in combination with fucoidan (ScF) from *S. cichorioides* or carboxymethylated derivative of fucoidan (ScFCM) on the viability and proliferation of human melanoma cells (SK-MEL-28). SK-MEL-28 cells were treated with (**a**) 2-DG (1 mM) in combination with ScF (50, 100, 200 µg/mL) or (**b**) 2-DG (1 mM) with ScFCM (50, 100, 200 µg/mL). Cell viability was estimated by the MTS assay. The data are presented as mean ± SD for triplicate experiments. A one-way ANOVA and Tukey’s HSD test for multiple comparisons indicated the statistical significance (*ns*—no significant difference, * *p* < 0.05, ** *p* < 0.01). (**c**) Median effect plot calculated using the CompuSyn software 1.0.1 software (ComboSyn, Inc., USA) showing the type of interactions of 2-DG with ScF or ScFCM (combination index (CI) is a quantitative measure of the degree of interaction between different treatments. CI values equal to 0.9–1.1 indicate an additive effect; CI values greater than 1.1 indicate antagonism; CI values less than 0.7 indicate synergism).

As shown in Figure 4a,b, 2-DG at a concentration of 1 mM inhibited the viability of SK-MEL-28 cells by 20% compared to non-treated cells (control).

ScF at concentrations of 50, 100, and 200 µg/mL enhanced the inhibitory effect of 2-DG (1 mM) on the SK-MEL-28 cell proliferation by 17, 20, and 38%, respectively, compared to the cells treated with 2-DG alone for 72 h (Figure 4a). While ScFCM (at 50, 100, and 200 µg/mL) in combination with 2-DG reduced the cell proliferation by 22, 31, and 51%, respectively, compared to the 2-DG-treated SK-MEL-28 cells (Figure 4b). The combined effect of 2-DG with ScF (CI = 0.196; 0.228; 0.146) or ScFCM (CI = 0.238; 0.256; 0.182) on SK-MEL-28 cells was synergistic, as confirmed by Chou and Talalay’s method for drug interactions (Figure 4c). Overall, it was shown that ScF and ScFCM in combination with 2-DG at low, non-toxic concentrations effectively suppressed the growth of SK-MEL-28 cells, with a greater extent of the carboxymethylated derivative of fucoidan ScFCM in combination with 2-DG.

### 2.4. The Metabolically Oriented Effect of Fucoidan from S. cichorioides and Its Carboxymethylated Derivative on the Colony Formation of Human Melanoma Cells

**Figure 5:**
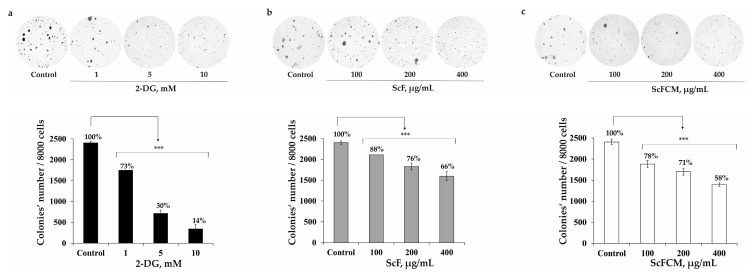
The inhibiting effect of 2-DG, fucoidan from *S. cichorioides* (ScF), and its carboxymethylated derivative (ScFCM) on colony formation in human melanoma cells (SK-MEL-28). SK-MEL-28 cells were treated with (**a**) 2-DG (1, 5, 10 mM), (**b**) ScF (100, 200, 400 µg/mL), and (**c**) ScFCM (100, 200, 400 µg/mL) in soft agar. The number of colonies was counted under a microscope (at a total magnification of 40×) using the ImageJ software version 1.50i bundled with 64-bit Java 1.6.0_24 (“NIH”, Bethesda, MD, USA). Results are presented as mean ± standard deviation (SD); *** *p* < 0.001.

Then we tested whether the fucoidan from *S. cichorioides* (ScF) or its carboxymethylated derivative (ScFCM) increased the inhibiting activity of 2-DG on the colony formation in human melanoma cells (SK-MEL-28) using the soft agar assay. First, we measured the effect of each of the investigated compounds separately (Figure 5a–c). 2-DG at only 1, 5, and 10 mM decreased the number of SK-MEL-28 colonies by 27, 70, and 86, respectively, compared to the control (Figure 5a). ScF at 100, 200, and 400 µg/mL inhibited the colony growth of SK-MEL-28 cells by 12, 24, and 34%, respectively (Figure 5b), while ScFCM at the same concentrations suppressed the colony formation in SK-MEL-28 cells by 22, 29, and 42%, respectively (Figure 5c). The effect of 2-DG, ScF, and ScFCM on colony formation in RPMI-7951 and SK-MEL-5 was also tested. The investigated polysaccharides were confirmed to possess a moderate inhibiting activity on the colony formation and growth of RPMI-7951 and SK-MEL-5 cells (Supplementary Figure S3a–f).

**Figure 6:**
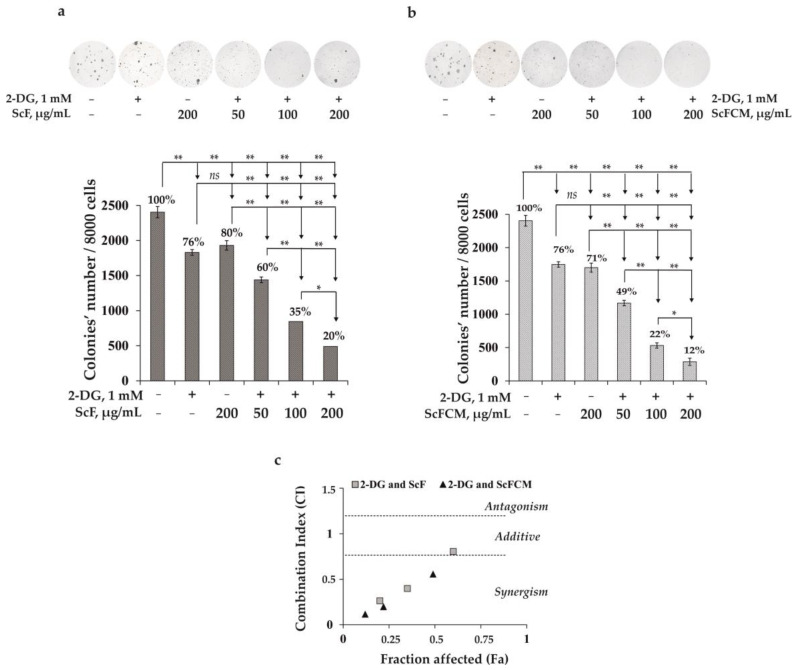
The metabolically oriented effect of 2-DG in combination with fucoidan from *S. cichorioides* (ScF) or carboxymethylated derivative of fucoidan (ScFCM) on the colony formation in human melanoma cells (SK-MEL-28). SK-MEL-28 cells were treated with (**a**) 2-DG (1 mM) in combination with ScF (50, 100, 200 µg/mL) or (**b**) 2-DG (1 mM) with ScFCM (50, 100, 200 µg/mL) in soft agar. The number of colonies was counted under a microscope (at a total magnification of 40×) using the ImageJ software version 1.50i bundled with 64-bit Java 1.6.0_24 (“NIH”, Bethesda, MD, USA). Results are presented as mean ± standard deviation (SD). A one-way ANOVA and Tukey’s HSD test for multiple comparisons indicated the statistical significance (*ns*—no significant difference, * *p* < 0.05, ** *p* < 0.01). (**c**) Median effect plot calculated using the CompuSyn software 1.0.1 software (ComboSyn, Inc., USA) showing the type of interactions of 2-DG with ScF or ScFCM.

Based on data of the colony-inhibiting effect for each of the investigated compounds (Figure 5), we treated SK-MEL-28 cells with 2-DG at a low dose of 1 mM in combination with ScF or ScFCM at concentrations of 50, 100, and 200 µg/mL. We found that ScF (50, 100, and 200 µg/mL) potentiated the inhibiting effect of 2-DG (1 mM) on colony formation in SK-MEL-28 cells by 16, 41, and 56%, respectively, compared to the cells treated with 2-DG only (Figure 6a). ScFCM at 50, 100, and 200 µg/mL in combination with 2-DG (1 mM) decreased the number of colonies by 27, 54, and 64%, respectively, compared to the 2-DG-treated cells (Figure 6b). The effects of the combinations, 2-DG with ScF (100 and 200 µg/mL) and 2-DG with ScFCM (50, 100, and 200 µg/mL), were defined as synergistic (Figure 6c). It should be noted that the effect from the treatment of SK-MEL-28 cells with ScFCM in combination with 2-DG was more pronounced than that from the treatment with the native fucoidan, ScF. It is worthwhile to notice that the treatment of RPMI-7951 and SK-MEL-5 melanoma cells by combinations of 2-DG with ScF or ScFCM at the same experimental conditions did not lead to significant inhibition of colonies formation in the tested cell lines (Supplementary Figure S4a–d).

### 2.5. The Molecular Mechanism of the Metabolically Oriented Effect of Fucoidan from S. cichorioides and Its Carboxymethylated Derivative

**Figure 7:**
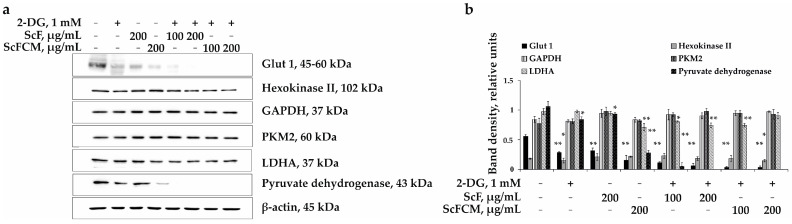
The metabolically oriented effect of 2-DG in combination with fucoidan from *S. cichorioides* (ScF) and carboxymethylated derivative of fucoidan (ScFCM) on the regulation of glycolysis enzymes in human melanoma cells (SK-MEL-28). (**a**) The regulation of expression of Glut 1, Hexokinase II, GAPDH, PKM2, LDHA, pyruvate dehydrogenase, and β-actin by 2-DG (1 mM) in combination with ScF (100 and 200 μg/mL) or ScFCM (100 and 200 μg/mL) in SK-MEL-28 cells. (**b**) Relative band density was measured using the Quantity One 1D analysis software version 4.6.7 (Bio-Rad, Hercules, CA, USA). Band density was normalized to β-actin total level. Results are presented as mean ± standard deviation (SD); * *p* < 0.05, ** *p* < 0.01.

In recent decades, research efforts have focused on targeting cancer cell metabolism since it strongly differs from glucose metabolism in normal cells [26]. In this study, we assessed the effect of an inhibitor of glycolysis, 2-DG, in combination with ScF or ScFCM on the activity of members of glycolysis by Western blot analysis (Figure 7a,b). The investigated compounds alone slightly influenced the expression of the glucose transporter Glut 1, while the combined treatment of SK-MEL-28 cells with 2-DG (1 mM) and ScF (100 and 200 μg/mL) or ScFCM (100 and 200 μg/mL) led to its complete down-regulation (Figure 7a,b). The expression of Hexokinase II, glyceraldehyde-3-phosphate-dehydrogenase (GAPDH), and pyruvate kinase M2 (PKM2) proteins seemed to be unaltered after treatment with the polysaccharides ScF and ScFCM (200 μg/mL) separately; however, ScF or ScFCM at 100 and 200 μg/mL enhanced the inhibiting effect of 2-DG (1 mM) on Hexokinase II and PKM2 activation. The GAPDH expression did not change under the same experimental conditions. The expression levels of lactate dehydrogenase A (LDHA) and pyruvate dehydrogenase, which control the pyruvate to lactate conversion and play a key role in glycolysis, cell growth, and tumor maintenance, proved to be significantly suppressed by combined treatment with 2-DG and ScF or ScFCM (Figure 7a,b), which led to the disruption of the metabolic pathway of SK-MEL-28 melanoma cells and the suppression of cell proliferation.

## 3. Discussion

Melanoma is a highly aggressive skin cancer with an increasing incidence, and the development of novel treatment strategies remains an urgent need [27]. Cancer cells that are phenotypically characterized by aerobic glycolysis prefer glucose uptake and lactate production, even in the presence of oxygen (Warburg effect), while glutamine is extremely important for oxidative phosphorylation (OXPHOS) and redox regulation [9,28,29]. Metabolic reprogramming (plasticity) in cancer is rarely static but, instead, a highly dynamic process that allows rapid adaptability, which requires both the flexibility to utilize different metabolic substrates and the ability to process metabolic substrates in different ways. It has been shown that metabolic reprogramming is a key driver of melanoma progression and response to current standard-of-care anticancer and immune therapies [30]. The inherent plasticity of melanoma cell metabolism has been evidenced by reversible metabolome alterations that occur during metastasis and response to anticancer therapies and in the diversity of fuel sources melanoma cells can utilize to survive in response to nutrient deprivation and exposure to different microenvironmental niches [9,31,32]. As such, this inherent metabolic plasticity creates a moving target for therapeutic interventions and consequently poses a major challenge to effective therapy.

The sulfated polysaccharides of brown algae, fucoidans, and their derivatives proved to exhibit potent anti-proliferative, anti-migratory, anti-metastatic, and metabolically oriented activities against human cancer cells and could be used as promising metabolic modifiers for increasing the effectiveness of melanoma therapy [16,33,34]. In the present study, we identified the fucoidan isolated from the brown alga *S. cichorioides* as almost pure fucan, consisting of a backbone of 1,3-linked α-L-fucopyranose residues with a small proportion of 1,4-linked α-L-fucopyranose residues. A small amount of single α-L-fucose residues were present in the branches at position 2. Sulfate groups occupied positions 2 and 4 of the fucopyranose residues. The fucoidan from *S. cichorioides* was carboxymethylated in order to improve its functional properties. Several studies have demonstrated that the chemical modifications of polysaccharides, such as sulfation, amination, phosphorylation, and selenization, significantly influence their structural composition, molecular weight, linkage pattern, and ionic characteristics of polysaccharides; as a result, the functional properties of polysaccharides are also modified [21,22,35,36].

We have found that the human melanoma cells tested (SK-MEL-28, RPMI-7951, and SK-MEL-5) differed in the ability to take up glucose and excrete the lactate and glutamate that might be related to the metabolic plasticity of cell lines even within one cancer type (Figure 1). SK-MEL-28 cells were determined to take up glucose and excrete lactate at a high rate but did not show the glutamate production characteristic of aerobic glycolysis (Figure 1b,e). RPMI-7951 cells were able to take up a high amount of glucose but produced smaller amounts of lactate and glutamate, and SK-MEL-5 cells were characterized by a low glucose uptake and lactate excretion but a high glutamate production (Figure 1c,d,f,g). In our work, we focused on the SK-MEL-28 melanoma cell line having aerobic glycolysis as a preferable metabolic pathway.

Thus, we have found for the first time that the fucoidan from *S. cichorioides* and its carboxymethylated derivative can decrease glucose uptake and lactate excretion in SK-MEL-28 melanoma cells in a dose-dependent manner (Figure 2). Previously, the effect of fucoidans from brown algae on glucose uptake was investigated in order to assess their anti-diabetic potential. It was demonstrated that the treatment with fucoidan from brown alga *Undaria pinnatifida* stimulated glucose uptake in normal 3T3 adipocytes and restored insulin-stimulated glucose uptake in obesity-induced insulin-resistant adipocytes [17]. Shan and co-authors [37] indicated the potential effects of fucoidan from *Ascophyllum nodosum* on the regulation of blood glucose levels by direct inhibition of glucose transport via SGLT1, causing the glucose transport to markedly reduce and relieving postprandial hyperglycemia.

2-Deoxy-D-glucose (2-DG), a glucose analog inhibiting glycolysis, is widely used as a metabolic modifier to disturb cancer cell proliferation [12]. However, the potential for using 2-DG as a single therapeutic agent appears to be limited by the development of toxicity, diaphoresis, hypoglycemia, and disturbances of the CNS through daily administrations of large doses of 2-DG over a long period of time [38]. Thus, we hypothesized that a combination of 2-DG and fucoidan or its carboxymethylated derivative could enhance the inhibition of melanoma cell proliferation at lower, non-toxic concentrations, thereby minimizing probable side effects of 2-DG and potentially helping to overcome the therapeutic resistance of melanoma cells.

2-DG, sulfated 1,3-linked α-L-fucan from brown alga *S. cichorioides* (ScF), and its carboxymethylated derivative (ScFCM) were separately shown to inhibit the viability, proliferation, and colony formation of melanoma cells to variable degrees (Figure 3 and Figure 4). Recently, it has been reported that 2-DG inhibited the viability and proliferation of human cancer cells via induction of apoptosis [11,39,40]. Our results on the anticancer activity of fucoidan from *S. cichorioides* are also consistent with the published data, which indicate that it significantly inhibited TPA-induced neoplastic transformation of mouse epidermal cells JB6 Cl41 [24,41]. In our study, we, for the first time, assessed the effect of the carboxymethylated derivative of ScFCM on the viability, proliferation, and colony formation of SK-MEL-28 cells. The anticancer activity of ScFCM was comparatively higher than that of the native polysaccharide, which can be explained by its increased molecular weight, solubility in water, decreased viscosity, and a change in the molar ratios of monosaccharides.

The results obtained in this study indicate that the fucoidan from the brown alga *S. cichorioides* and its carboxymethylated derivative at low, non-toxic concentrations have a synergistic metabolically-oriented effect in combination with 2-DG that causes a pronounced inhibition of the viability, proliferation, and colony formation of SK-MEL-28 melanoma cells (Figure 4, Figure 5 and Figure 6). In the recent decade, the combined effect of 2-DG with chemotherapeutic drugs has been intensively investigated in order to increase the efficiency of anticancer therapy. Thus, the combination of 2-DG and cisplatin was shown to enhance the cytotoxicity in human head and neck cancer FaDu cells by mechanisms involving oxidative stress [42]. Also, 2-DG in combination with fenofibrate, safely used for over 40 years to decrease blood cholesterol in patients, synergistically inhibited the viability of human melanoma NM2C5, osteosarcoma 143B, and breast adenocarcinoma SKBR3 cell lines through regulation of activities of the AMPK and mTOR proteins, which led to greater ER stress and apoptosis induction [43]. Additionally, it was found that the combined treatment with 2-DG and sorafenib, an inhibitor of tyrosine protein kinases such as VEGFR, PDGFR, and the Raf family kinases, increased apoptosis and inhibited colony formation of the hepatocellular carcinoma cell line Hep3B and Huh7 cells [44]. To the best of our knowledge, the present study is the first to emphasize the potential of the combination of 2-DG with fucoidan and its carboxymethylated derivative as an approach to cancer treatment.

Glycolysis is a metabolic pathway that converts glucose into pyruvate in the cytoplasm, which leads to the production of adenosine triphosphate (ATP). The entire pathway of glycolysis contains ten steps of chemical reactions, with each catalyzed by a specific enzyme [45]. After glucose is taken up by membrane glucose transporters (Glut), which are overexpressed in cancer cells, it is converted into glucose-6-phosphate by hexokinase II. Then phosphofructokinase (PFK) catalyzes the phosphorylation of fructose-6-phosphate into fructose-1,6-bisphosphate. Glyceraldehyde-3-phosphate-dehydrogenase (GAPDH) then converts glyceraldehyde-3-phosphate into 1,3-bisphosphoglycerate. The enzyme pyruvate kinase M2 (PKM2) catalyzes the irreversible phosphoryl group transfer from phosphoenolpyruvate to pyruvate, from which ATP is formed. Cancer cells switch to and depend on aerobic glycolysis for survival. Therefore, lactate dehydrogenase (LDH), which catalyzes the conversion of pyruvate to lactate, is the key enzyme for determining the glycolytic phenotype of cancer cells; as such, it could be utilized as a therapeutic target. The pyruvate dehydrogenase complex catalyzes the conversion of pyruvate and CoA into acetyl-CoA and CO_2_ in the presence of NAD^+^ [46]. It has been reported that 2-DG competes with glucose for transport into the cell and can competitively inhibit glucose transport. The expression of glucose transporters and glycolytic enzymes increases under hypoxia, which is characteristic of cancer cells; this, in turn, enhances the uptake of 2-DG by cancer cells compared to normal cells under aerobic conditions [47,48,49]. In our study, 2-DG, fucoidan (ScF), and its carboxymethylated derivative (ScFCM) separately influenced slightly the expression of the Glut 1 transporter at low, non-toxic concentrations, but the treatment of SK-MEL-28 cells with 2-DG in combination with ScF or ScFCM further enhanced the inhibitory effect (Figure 7).

After entering the cell, 2-DG is phosphorylated by Hexokinase II to 2-deoxy-D-glucose-6-phosphate (2-DG-6-P), but, unlike glucose, 2-DG-6-P cannot be further metabolized by phosphoglucose isomerase (PGI) into a 5-carbon ring [12]. The results obtained in our study show that the combination of 2-DG with fucoidan or its derivative down-regulate the activities of Hexokinase II, PKM2, LDHA, and pyruvate dehydrogenase, which are the major components of the glycolysis pathway (Figure 7). This leads to the inhibition of growth colony formation and, consequently, the death of SK-MEL-28 melanoma cells.

To conclude, our study has assessed the feasibility and efficacy of a combined application of the glycolysis inhibitor 2-DG and a natural sulfated polysaccharide from the brown alga *S. cichorioides* and its derivatives to treat the aggressive human melanoma cells SK-MEL-28 and inhibit their proliferation associated with high glycolysis. Further investigations of the metabolically oriented effect of the combination of 2-DG with fucoidan or its derivative in vivo are of particular scientific interest. This combined strategy, due to the effective inhibition of uncontrolled cell proliferation and the enhancement of the therapeutic effect, is expected to be used in melanoma therapy in the future.

## 4. Materials and Methods

### 4.1. Reagents

The organic solvents, inorganic salts, and acids used in the study were manufactured by Dia-m (Moscow, Russia). The sorbent for chromatography Macro-Prep DEAE was purchased from Bio Rad Laboratories, Inc. (Hercules, CA, USA). The phosphate-buffered saline (PBS), L-glutamine, penicillin/streptomycin solution (10,000 U/mL, 10 µg/mL), Minimum Essential Medium Eagle (MEM), and reference standards (mannose, rhamnose, glucose, galactose, xylose, and dextrans) were purchased from the Sigma-Aldrich company (St. Louis, MO, USA). The MTS reagent 3-[4,5-dimethylthiazol-2-yl]-2,5-diphenyltetrazolium bromide was purchased from Promega (Madison, WI, USA). The trypsin, fetal bovine serum (FBS), 2-deoxy-D-glucose (2-DG), and the protein marker PageRuler^TM^ Plus Prestained Protein Ladder were purchased from Thermo Fisher Scientific (Waltham, MA, USA).

The cell lysis buffer (10×), glycolysis antibody sampler kit #8337, antibodies against Glut 1, and horseradish peroxidase (HRP) conjugated secondary antibody from rabbit and mouse were purchased from Cell Signaling Technology (Danvers, MA, USA), and β-actin was purchased from the Sigma-Aldrich company (St. Louis, MO, USA).

### 4.2. Isolation and Chemical Modification of Fucoidan from the Brown Alga S. cichorioides

#### 4.2.1. Brown Alga

The sample of the brown alga *S. cichorioides* (Sc) was collected in August 2021 from Peter the Great Bay, Sea of Japan (Russia). Fresh algal biomass was powdered and pretreated with 70% aqueous ethanol (=1:10 *w*/*v*) at room temperature for 10 days. The defatted alga was air-dried.

#### 4.2.2. Isolation of Fucoidan from the Brown Alga *S. cichorioides*

The fucoidan (ScF) was isolated from the brown alga sample (100 g) via the earlier described method [24] with a yield of 4 g.

#### 4.2.3. Carboxymethylation of Fucoidan from the Brown Alga *S. cichorioides*

Carboxymethylation of ScF was performed via the previously described method with some modifications [50]. In brief, ScF (500 mg) was mixed with 21 mL isopropanol and stirred for 15 min at room temperature. Then the polysaccharide mixture was supplemented with 8 mL of 20% NaOH drop-wise and stirred at room temperature for 3 h. The carboxymethylated agent (4.4 g chloroacetic acid, 8 mL of 20% NaOH, and 21 mL isopropanol) was added under stirring at 60 °C for 4 h. The obtained solution was cooled to room temperature; the pH was adjusted to 7.0 with 0.5 M HCl. The product was dialyzed against H_2_O for 72 h. The sample was freeze-dried to obtain the carboxymethylated derivative of fucoidan (hereinafter referred to as ScFCM).

### 4.3. Determination of the Structural Characteristics of Fucoidan from the Brown Alga S. cichorioides and Its Carboxymethylated Derivative

#### 4.3.1. Content of Carbohydrates

The content of carbohydrates was determined via the method of Michel Dubois et al. [51]. The absorbance was measured at 490 nm on a Power Wave XS microplate reader (BioTek, Winooski, VT, USA). Glucose (1 mg/mL) was used as the reference standard.

#### 4.3.2. Content of Sulfate Groups

The content of sulfate groups was determined via the turbidimetric method after hydrolysis of the sulfated fucoidan and its derivative with 1N HCl [52]. The absorbance was measured at 360 nm on a Power Wave XS microplate reader (BioTek, Winooski, VT, USA). K_2_SO_4_ (1 mg/mL) was used as the reference standard.

#### 4.3.3. Monosaccharide Composition

The monosaccharide composition of ScF and ScFCM was determined via gas-liquid chromatography (GLC) after hydrolysis using 2 M TFA (6 h, 100 °C) and obtainment of alditol acetate derivatives.

#### 4.3.4. Molecular Weight

The molecular weight of the ScF and ScFCM was determined via size-exclusion chromatography (SEC) on an Agilent 1100 Series HPLC system (Agilent Technologies, Waldbronn, Germany) equipped with a refractive index detector and series-connected SEC columns, Shodex OHpak SB-805 HQ and OHpak SB-803 HQ (Showa Denko, Tokyo, Japan). Elution was performed in a 0.15 M NaCl aqueous solution at 40 °C with a flow rate of 0.4 mL/min. Dextrans of 5, 10, 50, 80, 250, 410, and 670 kDa were used as reference standards.

#### 4.3.5. Nuclear Magnetic Resonance (NMR) Spectroscopy

^13^C NMR spectra were obtained on an Avance DPX-500 NMR spectrometer (Bruker BioSpin Corporation, Billerica, MA, USA) at 35 °C with acetone as the internal standard (pulse sequence—zgpg, flip angle—90°, scans—128, relaxation delay—1.5 s, and acquisition time—0.38 s). The sample concentration was 15 mg of polysaccharide per 600 mL of D_2_O.

### 4.4. Determination of the Metabolically Oriented Effect of Fucoidan from the Brown Alga S. cichorioides and Its Carboxymethylated Derivative

#### 4.4.1. Preparation of Fucoidan and Its Carboxymethylated Derivative for Bioassays

ScF and ScFCM were dissolved in sterile PBS to prepare the stock solutions at a concentration of 40 mg/mL. Cells were treated with serially diluted polysaccharides (50, 100, 200, 400, and 800 µg/mL) (with the culture medium used as diluent).

The vehicle control was the cells treated with the equivalent volume of PBS for all of the presented experiments.

#### 4.4.2. Cell Lines and Culture Conditions

The cells of the human melanoma cell lines SK-MEL-28 (ATCC^®^ no. HTB-72™), RPMI-7951 (ATCC^®^ no. HTB-66™), and SK-MEL-5 (ATCC^®^ no. HTB-70™) were purchased from the American Type Culture Collection (Manassas, VA, USA). Human immortal keratinocyte HaCaT was provided by the Shared research facility “Vertebrate cell culture collection” (Saint-Petersburg, Russia).

Cells were cultured in MEM supplemented with 10% heat-inactivated FBS and 1% penicillin/streptomycin solution at 37 °C in a humidified atmosphere containing 5% CO_2_. The number of passages was carefully controlled, and mycoplasma contamination was monitored on a regular basis.

#### 4.4.3. MTS Assay

The HaCaT (1.0 × 10^4^/200 µL) and SK-MEL-28 (0.8 × 10^4^/200 µL) cells were seeded in a 96-well plate and incubated for 24 h at 37 °C in a CO_2_ incubator.

To determine the concentration at which the compounds exert half of their maximal inhibitory effect on cell viability (IC_50_), the cells were treated with either PBS (vehicle control) or 2-deoxy-D-glucose (2-DG) at concentrations of 0.1, 1, 5, 10, and 20 mM, or ScF or ScFCM at concentrations of 100, 200, 400, and 800 µg/mL for 24, 48, and 72 h. The cells were subsequently incubated with 15 µL of the MTS reagent for 3 h. The absorbance of each well was measured at 490/630 nm on a Power Wave XS microplate reader (BioTek, Winooski, VT, USA).

To determine the effect of ScF and ScFCM on the proliferation of cancer cells in the metabolically suppressed conditions, the HaCaT and SK-MEL-28 cells were pretreated with 2-DG (1 mM) for 24 h, followed by treatment with ScF or ScFCM at 50, 100, and 200 µg/mL for additional 48 h. The cells were subsequently incubated with 15 µL of the MTS reagent for 3 h. The absorbance of each well was measured at 490/630 nm on a Power Wave XS microplate reader (BioTek, Winooski, VT, USA).

#### 4.4.4. Soft Agar Assay

The SK-MEL-28, RPMI-7951, and SK-MEL-5 cells (2.4 × 10^4^/mL) were treated with 2-DG (1, 5, and 10 mM) or ScF or ScFCM (100, 200, 400 µg/mL) separately; in the other experiments, the cells were treated with 2-DG (1 mM) in combination with ScF (50, 100, 200 µg/mL) or ScFCM (50, 100, 200 µg/mL). Then the cells were placed onto 0.3% BME agar containing 10% FBS, 2 mM L-glutamine, and 25 µg/mL gentamicin. The cultures were maintained at 37 °C in a 5% CO_2_ incubator for 14 days. The number of colonies was counted under an AE 20 Motic microscope using the ImageJ software version 1.50i bundled with 64-bit Java 1.6.0_24 (NIH, Bethesda, MD, USA).

#### 4.4.5. Glucose Uptake Assay

The HaCaT cells (5.0 × 10^4^/mL) and SK-MEL-28, RPMI-7951, and SK-MEL-5 cells (2.0 × 10^4^/mL) were seeded in a 24-well plate and cultured at 37 °C in a 5% CO_2_ incubator for 24 h. Then the cells were treated with either PBS (vehicle control) or ScF or ScFCM at concentrations of 50, 100, and 200 µg/mL for 24 h. The cells were then washed twice with PBS and starved in 1 mL of serum-free medium overnight. Afterward, they were washed thrice with PBS, glucose-starved by plating with 1 mL of the Krebs-Ringer-Phosphate-HEPES (KRPH) buffer (20 mM HEPES, 5 mM KH_2_PO_4_, 1 mM MgSO_4_, 1 mM CaCl_2_, 136 mM NaCl, and 4.7 mM KCl, pH 7.4, 2% BSA) for 40 min, and then stimulated with insulin (1 mM) for 20 min. Then 2-deoxy-D-glucose (2-DG) (10 mM) was added, and the cells were incubated for an additional 20 min. The cells were lysed by extraction buffer, the cell lysate was collected from the wells, and glucose uptake was measured using a Glucose Uptake Colorimetric Assay Kit (Sigma-Aldrich, St. Louis, MO, USA) according to the manufacturer’s instructions. The absorbance was measured at 412 nm on a Power Wave XS microplate reader (BioTek, Winooski, VT, USA) maintained at 37 °C. 2-Deoxy-D-glucose 6-phosphate (2-DG6P) (0.01 mM) was used as the reference standard.

#### 4.4.6. Lactate and Glutamate Production Assay

The SK-MEL-28, RPMI-7951, and SK-MEL-5 cells (2.5 × 10^4^/mL) were seeded in a 96-well plate and cultured at 37 °C in a 5% CO_2_ incubator for 24 h. Then the cells were treated with PBS or ScF at concentrations of 50, 100, and 200 µg/mL for 48 h. The culture medium was collected and used to determine the level of lactate production by the Lactate-Glo^TM^ Assay (Promega, Fitchburg, WI, USA) according to the manufacturer’s protocol. The cells remaining attached were washed twice with PBS and used to determine the level of glutamate production by the Glutamate-Glo^TM^ Assay (Promega, Fitchburg, WI, USA) according to the manufacturer’s protocol. The level of lactate and glutamate content was determined by the luminescence method on a PHERAstar FS multi-mode microplate reader (BMG Labtech, Offenburg, Germany) using the appropriate calibration curves.

#### 4.4.7. Western Blot Assay

The SK-MEL-28 cells (1.0 × 10^5^/mL) were seeded in 100 mm dishes and incubated for 24 h at 37 °C in a CO_2_ incubator. The cells were treated with 2-DG (1 mM) for 24 h and then with ScF or ScFCM at 100 and 200 µg/mL for an additional 48 h. The preparations of the cell lysate and the Western blot assay were previously described in the study by Vishchuk et al. [53]. In brief, the protein content was determined by the DC protein assay (Bio-Rad, Hercules, CA, USA). Lysates of protein (20–40 µg) were exposed to 10% or 12% SDS-PAGE and electrophoretically transferred to polyvinylidene difluoride membranes (PVDF) (Millipore, Burlington, MA, USA). The membranes were blocked with 5% non-fat milk (Bio-Rad) for 1 h and then incubated with the respective specific primary antibody at 4 °C overnight. Protein bands were visualized using an enhanced chemiluminescence reagent (ECL) (Bio-Rad, Hercules, CA, USA) after hybridization with an HRP-conjugated secondary antibody. The band density was quantified using the Quantity One 1D analysis software, version 4.6.7 (Bio-Rad).

#### 4.4.8. Statistical Analysis

All the assays were performed in at least triplicate. The results were expressed as mean ± standard deviation (SD). The obtained data were statistically processed by the one-way analysis of variance (ANOVA) and Tukey’s HSD test with significance levels of * *p* < 0.05 and ** *p* < 0.01.

## Figures and Tables

**Table 1:** Characteristics of the structure of fucoidan from *S. cichorioides* (ScF) and its carboxymethylated derivative (ScFCM).

Fraction	Yield, % *	Mw, kDa (Mw/Mn)	Content, % **		Monosaccharide Composition, mol %					
			Carbohydrate	SO_3_Na	Fuc	Gal	Man	Xyl	Rha	Glc
ScF	2.2 *	554 (2.8)	50	35	100	0	0	0	0	0
ScFCM	80 **	251 (2.4)	60	n.d.	100	0	0	0	0	0

* % of dried defatted alga weight; ** % of sample weight taken for experiment; n.d., not determined.

## Data Availability

The data presented in this study are available on request from the corresponding author.

## Supplementary Materials

The supporting information can be downloaded at: https://www.mdpi.com/article/10.3390/ijms241512050/s1.
