# Supplementary material for: The Combined Metabolically Oriented Effect of Fucoidan from the Brown Alga Saccharina cichorioides and Its Carboxymethylated Derivative with 2-Deoxy-D-Glucose on Human Melanoma Cells
Source: Int J Mol Sci. 2023 Jul 27;24(15):12050. doi: 10.3390/ijms241512050 (PMC10418387; doi:10.3390/ijms241512050)
Supplement: Supplementary file 1 [file ijms-24-12050-s001.zip › ijms-2497403-supplementary.pdf]

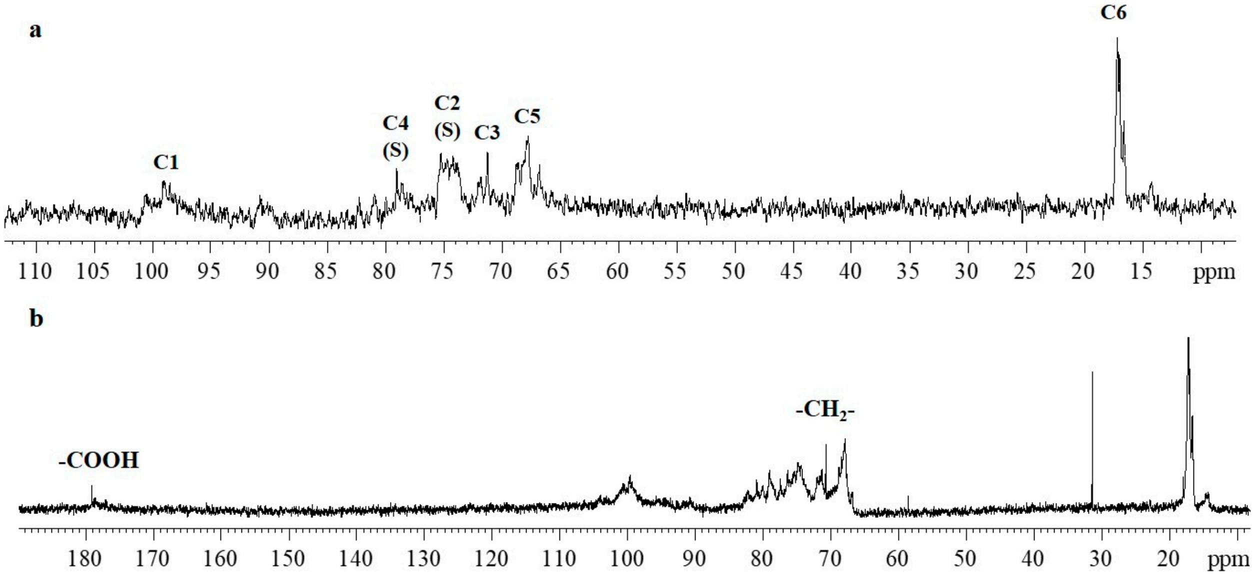

Supplementary Figure S1. The ^{13}C NMR spectra of (**a**) fucoidan ScF from *S. cichorioides* (signals C1–C6 of α-(1→3)-L-fucopyranose residues of the main chain are indicated) and (**b**) its carboxymethylated derivative ScFCM.

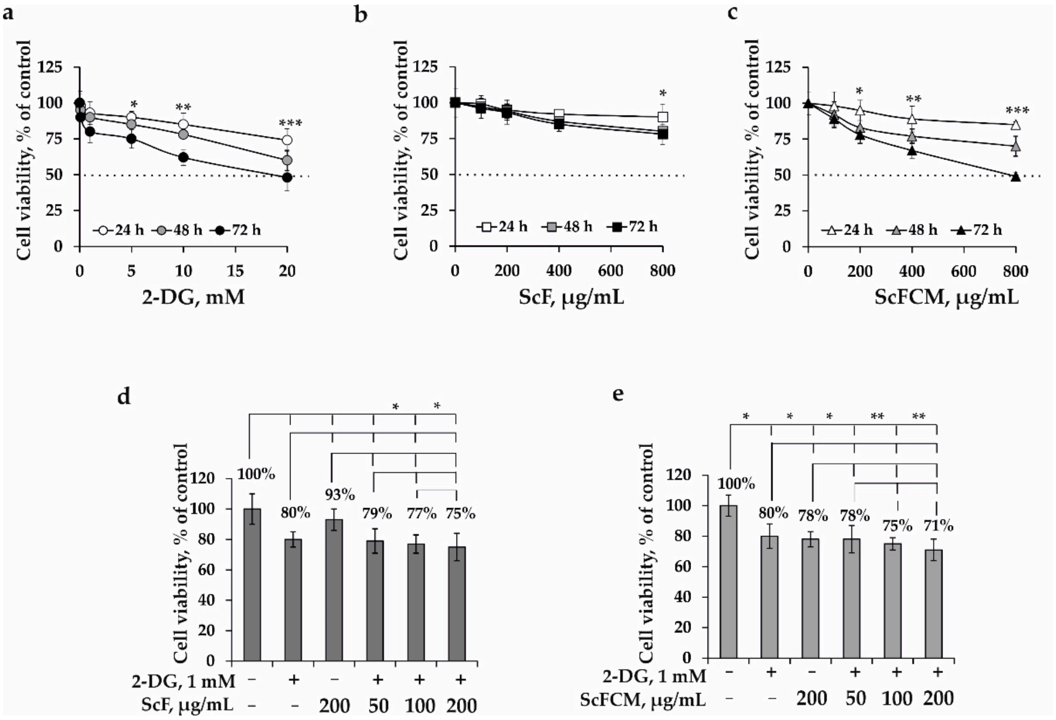

Supplementary Figure S2. The effect of 2-DG, the fucoidan (ScF) from *S. cichorioides*, and its carboxymethylated derivative (ScFCM) or their combinations on the viability and proliferation of human immortal keratinocyte (HaCaT).

HaCaT cells were treated by (**a**) 2-DG (0.1–20 mM), (**b**) ScF (100–800 μg/mL), (**c**) ScFCM (100–800 μg/mL), and incubated for 24, 48, and 72 h, (d) 2-DG (1 mM) in combination with ScF (50, 100, 200 μg/mL) or by (**e**) 2-DG (1 mM) in combination with ScFCM (50, 100, 200 μg/mL) and incubated for 72 h. The cell viability was estimated by the MTS assay. Data are presented as mean ± SD for triplicate experiments, *$p < 0.05$, **$p < 0.01$, and ***$p < 0.001$.

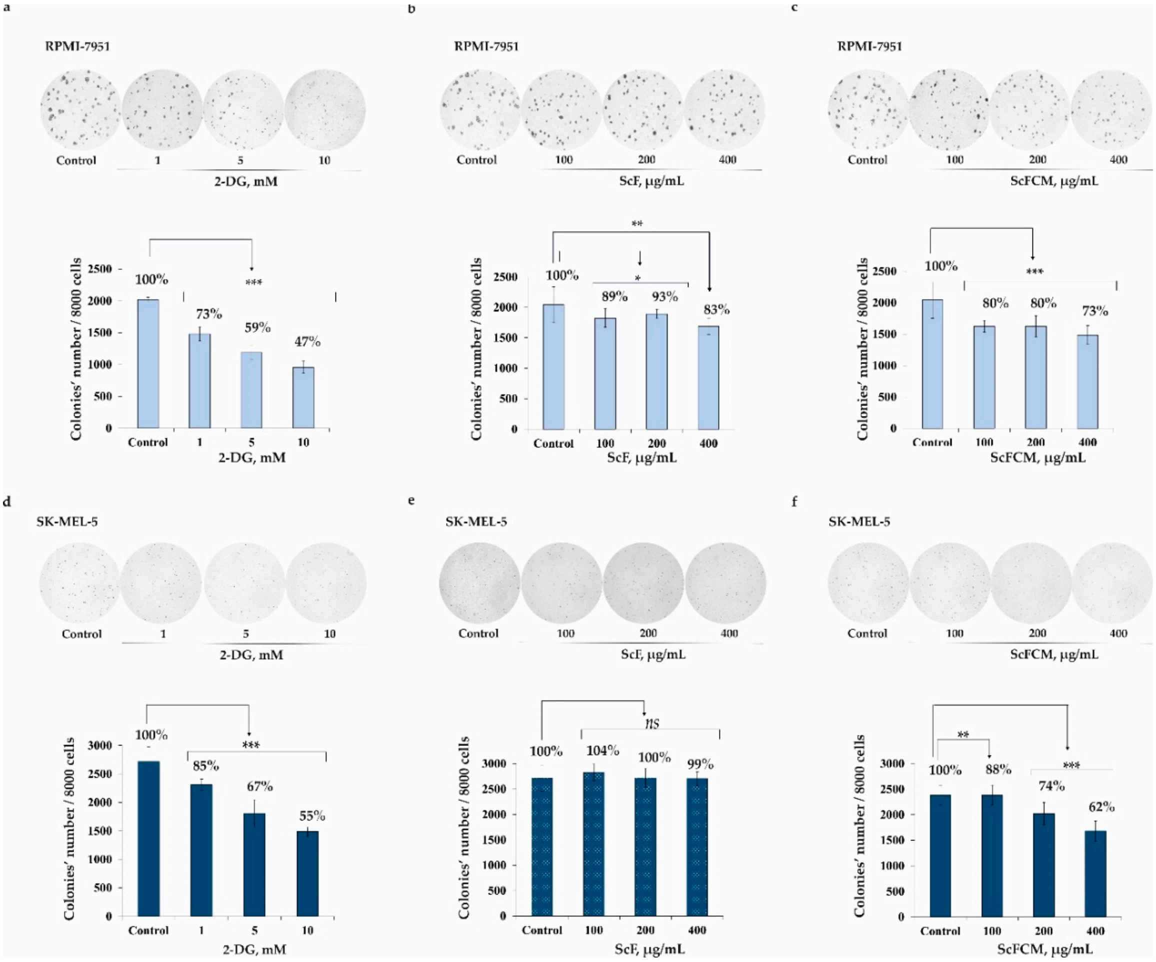

Supplementary Figure S3. The inhibiting effect of 2-DG, fucoidan from *S. cichorioides* (ScF), and its carboxymethylated derivative (ScFCM) on the colony formation in human melanoma cells (RPMI-7951 and SK-MEL-5).

RPMI-7951 and SK-MEL-5 cells were treated with (**a, d**) 2-DG (1, 5, 10 mM), (**b, e**) ScF (100, 200, 400 μg/mL), and (**c, f**) ScFCM (100, 200, 400 μg/mL) in soft agar. Number of colonies was counted under a microscope (at a total magnification of 40×) using the ImageJ software. Results are presented as mean ± standard deviation (SD); *ns* – not significant difference, $*p < 0.05$, $**p < 0.01$, and $***p < 0.001$.

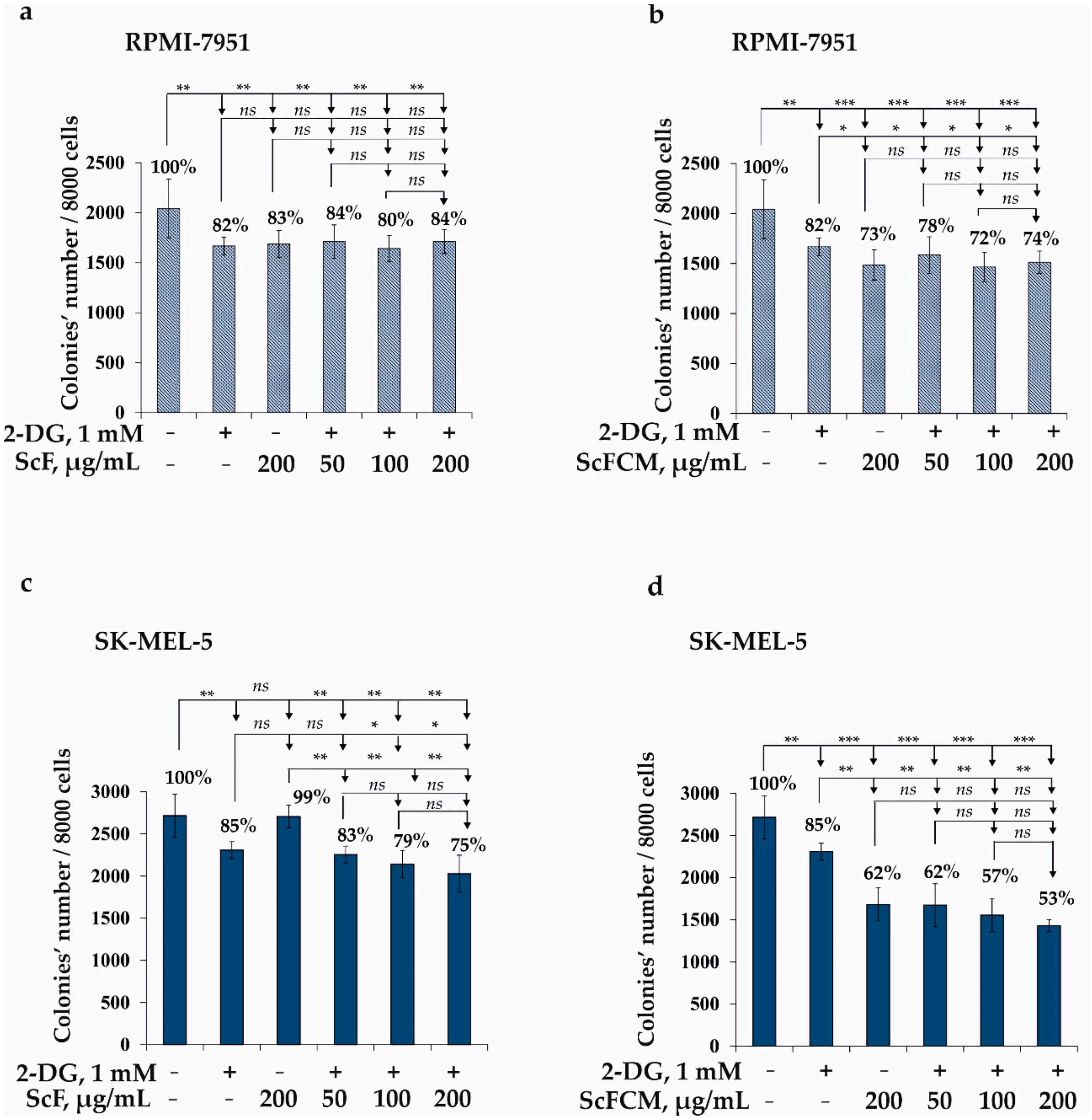

Supplementary Figure S4. The metabolically-oriented effect of 2-DG in combination with fucoidan from *S. cichorioides* (ScF) or carboxymethylated derivative of fucoidan (ScFCM) on the colony formation in human melanoma cells (RPMI-7951 and SK-MEL-5).

RPMI-7951 and SK-MEL-5 cells were treated with (**a, c**) 2-DG (1 mM) in combination with ScF (50, 100, 200 μg/mL) or (**b, d**) 2-DG (1 mM) with ScFCM (50, 100, 200 μg/mL) in soft agar. The number of colonies was counted under a microscope (at a total magnification of 40×) using the ImageJ software. Results are presented as mean ± standard deviation (SD); *ns* – not significant difference, *$p < 0.05$, **$p < 0.01$, and ***$p < 0.001$.
